# Bibliometric analysis of studies on stress urinary incontinence surgery

**DOI:** 10.1016/j.heliyon.2023.e21833

**Published:** 2023-11-04

**Authors:** Shufei Zhang, Jianfeng Liu, Shasha Hong, Lian Yang, Hanyue Li, Li Hong

**Affiliations:** Department of Obstetrics and Gynecology, Renmin Hospital of Wuhan University, Wuhan, 430060, Hubei Province, PR China

**Keywords:** Stress urinary incontinence, Bibliometric, Surgery, Midurethral sling, Mesh

## Abstract

**Background:**

Stress urinary incontinence (SUI) is characterized by the involuntary leakage of urine during activities that increase abdominal pressure. In recent years, a considerable number of studies on SUI surgery have been published. However, there has been a lack of systematic quantification and comprehensive summarization of these studies. Bibliometrics is a discipline that utilizes measurement methods to quantify scientific literature. Thus, this study utilized publications from the Web of Science (WOS) as a data source and conducted a comprehensive analysis and visualization of studies related to SUI surgery in recent years using bibliometric techniques.

**Methods:**

We conducted a search and retrieved information on 988 studies related to SUI surgery in the WOS Core Collection. The data covered ten years from September 7, 2013, to September 7, 2023. We employed VOSviewer software, CiteSpace software, and Bibliometrix for analysis and visualization.

**Results:**

Over the ten years, the number of publications exhibited a fluctuating trend, initially decreasing and then increasing. The United States emerged as the leading contributor in terms of both publication volume and quality. The University of Alabama Birmingham ranked as the institution with the highest number of publications, while the International Urogynecology Journal featured the most publications among journals.

**Conclusions:**

This paper presents a bibliometric analysis of publications related to SUI surgery from 2013 to 2023. The aim is to offer researchers a concise overview of the field and inspire future research directions.

## 1 Introduction

Pelvic floor disorders encompass a range of conditions resulting from deficiencies in the supportive structures of the pelvic floor. One significant symptom is stress urinary incontinence (SUI), characterized by the involuntary leakage of urine during activities that increase abdominal pressure, such as coughing, laughing, or sneezing [1]. Epidemiological surveys have indicated that SUI affects approximately 46 % of adult women, with its prevalence increasing with age [2]. While mild cases may be managed with Pelvic Floor Muscle Training and electrical stimulation, severe SUI often necessitates surgical intervention.

The common surgical options for SUI include Burch colposuspension, Autologous fascia pubovaginal sling, Midurethral sling (synthetic), and Bulking agents. If Midurethral sling (synthetic) is chosen, retropubic, transobturator, or single-incision sling may be chosen [3]. Among these, Midurethral sling (synthetic) is considered the preferred choice, although clinicians must carefully consider the specific risks and benefits associated with mesh, as well as explore alternative sling options. Despite the significant number of studies published in recent years on SUI surgery, there is a dearth of systematic quantification and summarization, leading to a wastage of scientific resources and hindrance to new scholars seeking a rapid understanding of the field.

Bibliometrics, as a discipline employing econometric methods, quantifies scientific literature and utilizes statistical and computer science techniques to comprehensively analyze countries, authors, journals, and citations. It evaluates the quantity and research hotspots of literature, enabling a quick and objective overview of the current development in a particular field and facilitates the identification of potential research trends and directions [4,5]. In this study, we employed publications from the Web of Science (WOS) as data sources and conducted a comprehensive bibliometric analysis and visualization of SUI surgery-related studies.

## 2 Materials and methods

### 2.1 Data acquisition

To comprehensively retrieve studies related to stress urinary incontinence (SUI) surgery, a search was conducted on September 8, 2023, in the WOS Core Collection. The search formula was ((TS = (stress urinary incontinence or urinary stress incontinence or stress incontinence)) AND ALL = (female or woman or women)) AND ALL = (Burch colposuspension or sling or tape or surgery) and Article (Document Types) and Retracted Publication (Exclude – Document Types) and Proceeding Paper (Exclude – Document Types) and Early Access (Exclude – Document Types) and English (Languages).

**Fig. 1:**
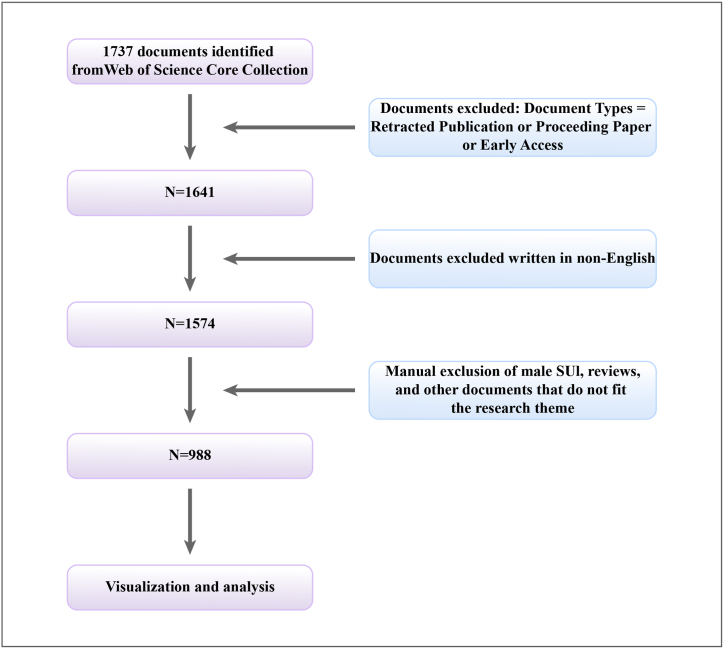
Flow chart of data screening.

To ensure the study's novelty and timeliness, our search spanned ten years from September 7, 2013, to September 7, 2023. The entire literature search and manual screening process were completed within one day. Initially, 1574 results were obtained from the search. After manual screening, excluding non-English articles, studies on male SUI, reviews, and other irrelevant literature, a total of 988 relevant documents were collected. For subsequent analysis, we extracted detailed information for each document, such as title, keywords, authors, country, affiliation, year of publication, references, and citations (Fig. 1).

### 2.2 Bibliometric analysis and visualization

Microsoft Office Excel 2019 was used to analyze annual publications and to visualize trends in publications, which we temporarily excluded from this analysis due to less than one year of data for 2013 and 2023. Using VOSviewer (Version 1.6.19.0, Leiden University, the Netherlands), Bibliometrix, and CiteSpace for bibliometric analysis and visualization [[6], [7], [8]]. VOSviewer is a powerful bibliometric analysis tool that enables the examination of authors, journals, organizations, citations, and co-citation relationships. Bibliometrix (https://www.bibliometrix.org) is based on the "bibliometrix" package in the R environment, offering comprehensive science mapping analysis by mapping authors, countries, organizations, and publications between countries. CiteSpace, developed by Prof. Chaomei Chen, is an information visualization software specifically designed for bibliometric analysis. It supports various data sources, performs data preprocessing and cleaning, and aids researchers in uncovering relationships and trends within academic literature. CiteSpace utilizes multiple analytical methods such as burst word analysis, temporal analysis, and cluster analysis to explore the correlation between literature and the evolution of research hotspots. It is an invaluable tool for researchers seeking to understand the relationships and trends within academic literature and the evolution of research hotspots.

## 3 Results

### 3.1 Global publication trends on SUI surgery

**Fig. 2:**
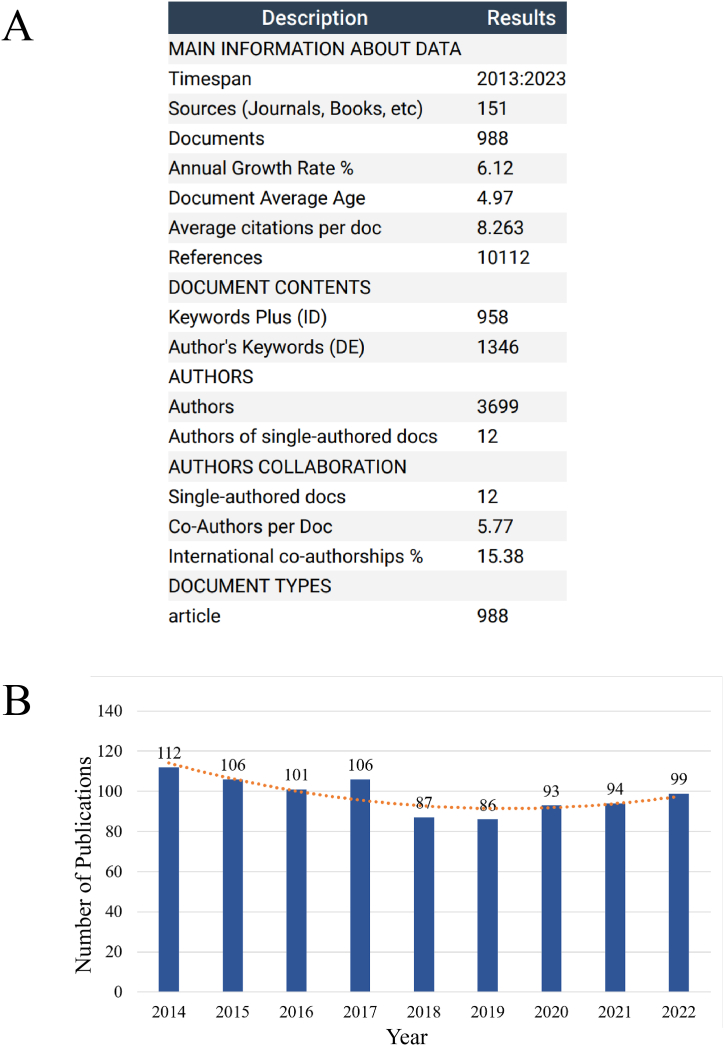
(A) Overview of publications related to SUI surgery; (B) The overall trend in the growth of publications about SUI surgery around the world from 2014 to 2022. The curve shows the fitting curves of growth trends in publications.

According to our search strategy, a total of 988 publications related to SUI surgery research were obtained from the WOS Core Collection. These publications were published in 151 different journals between the years 2013 and 2023. Among these publications, the majority were co-authored, while only 12 publications were sole-authored (Fig. 2A).

When examining the annual publication trends from 2014 to 2022, there was a fluctuating pattern, with a decrease followed by an increase in the number of publications (Fig. 2B). The steady increase in publications in recent years suggests that researchers are increasingly focusing on SUI surgery-related research. However, it is important to note that the total number of articles alone indicates that further research on SUI surgery is still insufficient.

### 3.2 Contribution of countries and institutions on SUI surgery

**Fig. 3:**
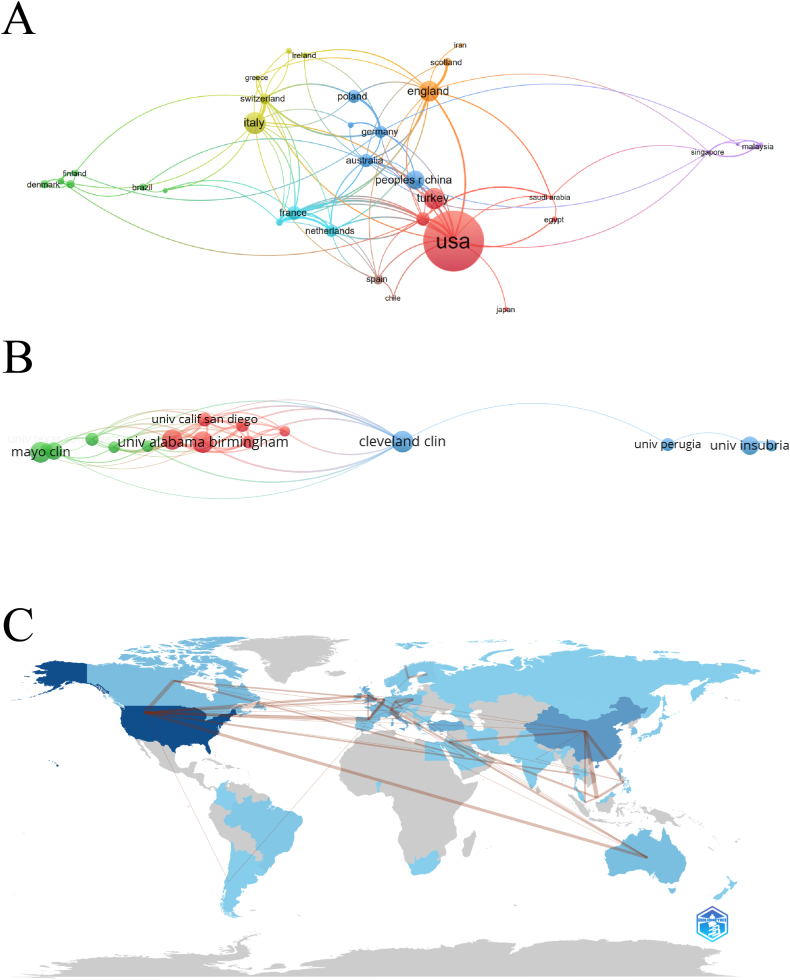
(A) Network visualization map for countries based on the number of publications in this field (N = 36, minimum number of publications of one country ≥5); (B) Network visualization map for institutions based on the number of publications in this field (N = 23, minimum number of publications of one country ≥10); (C) The intensity of cooperation in this field among countries around the world (The thickness of the red line representing the intensity of cooperation).

A total of 57 countries worldwide have made significant contributions to the field of SUI surgery. Among them, the United States had the highest number of articles, with 295 publications (29.86 %) over ten years. These publications received a total of 2762 citations, averaging 9.36 citations per article (Table 1, Fig. 3A). China, on the other hand, ranked second with 118 articles (11.94 %), 512 citations in total, and an average of 4.34 citations per article. Italy followed with 72 articles (7.29 %), Turkey with 68 articles (6.88 %), and England with 65 articles (6.58 %). Examining the top ten countries in terms of publication numbers reveals that Australia, despite ranking last, had the highest average number of citations (13.85). Conversely, China, the second-ranked country, had the lowest average number of citations among the top ten countries, suggesting that the research quality may be comparatively lower than in other countries.

**Table 1:** The top 10 countries with the largest numbers of documents.

Rank	Country	Documents (n)	Percentage (n/988)	Citations	Average Citation
1	USA	295	29.86	2762	9.36
2	China	118	11.94	512	4.34
3	Italy	72	7.29	730	10.14
4	Turkey	68	6.88	343	5.04
5	England	65	6.58	820	12.62
6	France	38	3.85	333	8.76
7	Canada	37	3.74	406	10.97
8	South Korea	37	3.74	203	5.49
9	Poland	35	3.54	288	8.23
10	Australia	34	3.44	471	13.85

Globally, 1411 institutions published relevant research in the field of SUI surgery during the decade. However, only 23 institutions had more than 10 publications, aligning with the country-level analysis. Six out of the top ten institutions were located in the United States. The University of Alabama Birmingham, Chang Gung University, and Cleveland Clinic had the highest number of publications, each with 23, and average citations of 18.74, 6.83, and 13, respectively (Table 2). Conducting a collaborative relationship analysis between institutions and countries revealed that top-ranked institutions maintained close cooperation with each other, including the University of Alabama Birmingham and Cleveland Clinic, as well as the University of Pittsburgh. However, highly ranked Asian institutions with the highest publication numbers, such as Chang Gung University and Chang-Gung Memorial Hospital, did not exhibit collaborative relationships with other institutions. This suggests that the scope of global SUI surgery research remains relatively limited (Fig. 3B). Furthermore, this phenomenon is also reflected at the national level, with Europe, the United States, and Asian countries demonstrating a relatively fragmented development pattern (Fig. 3C).

**Table 2:** The top 10 institutions with the largest numbers of documents.

Rank	Institutions	Country	Documents (n)	Percentage (n/988)	Citations	Average Citation
1	University of Alabama Birmingham	USA	23	2.33	431	18.74
2	Chang Gung University	China	23	2.33	157	6.83
3	Cleveland Clinic	USA	23	2.33	299	13
4	Mayo Clinic	USA	22	2.23	352	16
5	University of Pittsburgh	USA	21	2.13	387	18.43
6	University of Insubria	Italy	19	1.92	239	12.58
7	Vanderbilt University	USA	18	1.82	338	18.78
8	Chang-Gung Memorial Hospital	China	17	1.72	126	7.41
9	University of California, San Diego	USA	14	1.42	328	23.43
10	University of Aberdeen	England	14	1.42	295	21.07

### 3.3 Contribution of journals and co-cited journals on SUI surgery and core literature

**Fig. 4:**
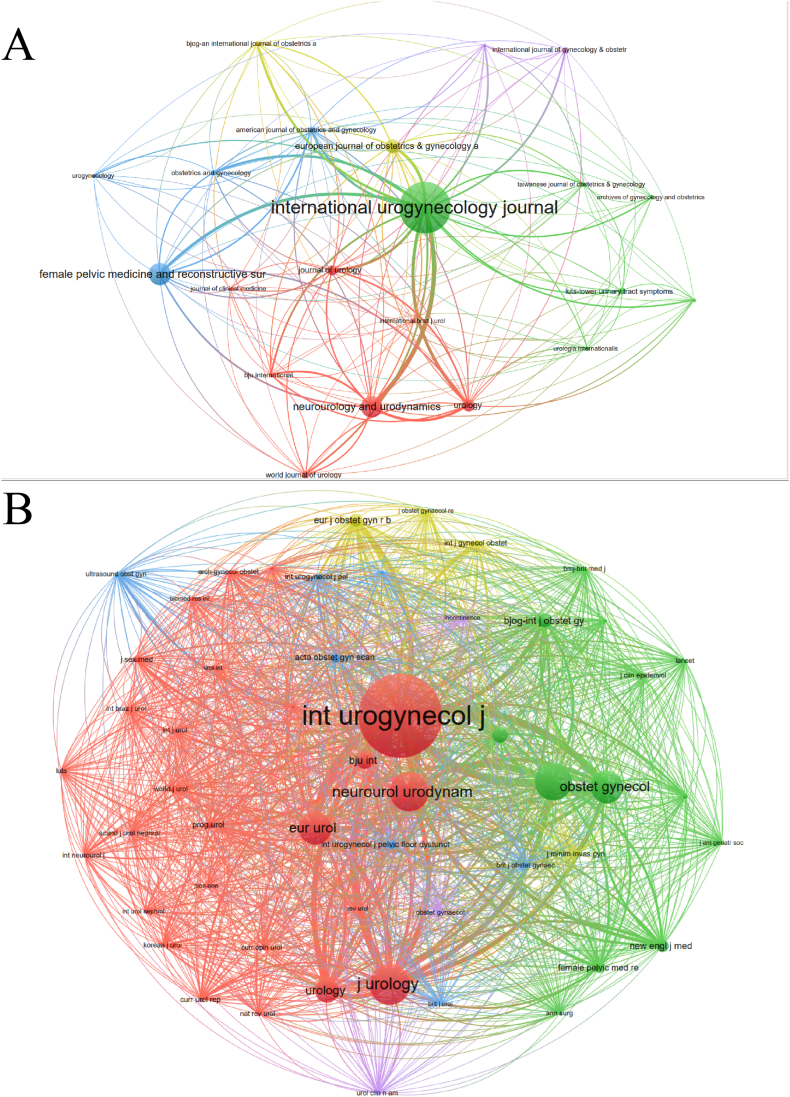
(A) Network visualization map for journals based on the frequency of citations (N = 21, minimum number of citations of one journal ≥10); (B) Network visualization map for journals based on the frequency of co-citation (N = 53, minimum number of citations of one journal ≥50).

Since September 7, 2013, studies related to SUI surgery have been published in a total of 151 journals, with 41 of them publishing more than five papers. Given that SUI is a urogynecologic disorder, a majority of the studies were focused on journals in the fields of urology and obstetrics & gynecology. Interestingly, the top 10 publications were found in journals specializing in urogynecology. The three leading journals in terms of SUI surgery publications are the *International Urogynecology Journal*, *Female Pelvic Medicine and Reconstructive Surgery*, and *Neurourology and Urodynamics*, with 206, 79, and 77 articles, respectively. It is worth noting that the *American Journal of Obstetrics and Gynecology*, with an average citation of 25.47 and a current impact factor of 9.8, is considered one of the most authoritative journals in the field of obstetrics and gynecology. However, as a whole, journals in this field have a relatively low impact factor and lack significant influence (Table 3, Fig. 4A).

**Table 3:** The top 10 journals with the largest numbers of documents and the highest frequency of co-citation.

Rank	Journals	Documents (n)	Citations	Average Citation	Co-cited journals	Co-citations
1	International Urogynecology Journal	206	1682	8.17	International Urogynecology Journal	3909
2	Female Pelvic Medicine and Reconstructive Surgery	79	452	5.72	Journal of Urology	1669
3	Neurourology and Urodynamics	77	728	9.45	Neurourology and Urodynamics	1587
4	European Journal of Obstetrics & Gynecology and Reproductive Biology	47	315	6.70	American Journal of Obstetrics and Gynecology	1500
5	Urology	41	439	10.71	European Urology	1288
6	Journal of Urology	33	461	13.97	Obstetrics and Gynecology	1280
7	World Journal of Urology	24	225	9.38	Urology	898
8	LUTS-Lower Urinary Tract Symptoms	20	82	4.1	BJU International	628
9	American Journal of Obstetrics and Gynecology	17	433	25.47	Cochrane Database of Systematic Reviews	550
10	International Journal of Gynecology & Obstetrics	16	69	4.31	BJOG - An International Journal of Obstetrics and Gynaecology	514

In our screening process, we identified 53 journals with more than 50 co-citations. Among them, the *International Urogynecology Journal* had the highest number of co-citations (3,909), followed by the *Journal of Urology* (1,669) and *Neurourology and Urodynamics* (1,587) (Fig. 4B). This indicates that the *International Urogynecology Journal* is the most influential journal in the field of SUI surgery. However, due to its focus on urogynecological disorders, it is categorized as Q2 according to Journal Citation Reports (JCR) and has an impact factor of only 1.8.

We have also compiled a list of some highly regarded articles, among which Megan O. Schimpf et al.'s “Sling surgery for stress urinary incontinence in women: a systematic review and meta-analysis" received 165 citations”. This demonstrates the high level of interest among researchers worldwide in cohort studies and comprehensive studies (Table 4).

**Table 4:** The top 10 documents with the largest numbers of citations.

Rank	Title	DOI	First Author	Journal	PMID	Publication year	Citations
1	Sling surgery for stress urinary incontinence in women: a systematic review and metaanalysis	10.1016/j.ajog.2014.01.030	Megan O Schimpf	American Journal of Obstetrics and Gynecology	24487005	2014	165
2	Surgery versus physiotherapy for stress urinary incontinence	10.1056/NEJMoa1210627	Julien Labrie	New England Journal of Medicine	24047061	2013	110
3	Adverse events after first, single, mesh and non-mesh surgical procedures for stress urinary incontinence and pelvic organ prolapse in Scotland, 1997–2016: a population-based cohort study	10.1016/S0140-6736(16)32572-7	Joanne R Morling	Lancet	28010993	2017	98
4	Complications following vaginal mesh procedures for stress urinary incontinence: an 8 year study of 92,246 women	10.1038/s41598-017-11821-w	Kim Keltie	Scientific Reports	28931856	2017	97
5	5-year longitudinal followup after retropubic and transobturator mid urethral slings	10.1016/j.juro.2014.08.089	Kimberly Kenton	Journal of Urology	25158274	2015	90
6	Removal or Revision of Vaginal Mesh Used for the Treatment of Stress Urinary Incontinence	10.1001/jamasurg.2015.2590	Blayne Welk	JAMA Surgery	26352538	2015	71
7	Pelvic Floor Disorders Network. A model for predicting the risk of de novo stress urinary incontinence in women undergoing pelvic organ prolapse surgery	10.1097/AOG.0000000000000094	J Eric Jelovsek	Obstetrics and Gynecology	24402598	2014	53
8	Long-term efficacy of the transobturator and retropubic midurethral slings for stress urinary incontinence: single-center update from a randomized controlled trial	10.1016/j.eururo.2014.04.004	Elisabetta Costantini	European Urology	24768493	2014	53
9	Five-year results of a randomized trial comparing retropubic and transobturator midurethral slings for stress incontinence	10.1016/j.eururo.2014.01.031	Eija Laurikainen	European Urology	24508070	2014	51
10	Long-term follow-up of a multicentre randomised controlled trial comparing tension-free vaginal tape, xenograft and autologous fascial slings for the treatment of stress urinary incontinence in women	10.1111/bju.12851	Zainab A Khan	BJU International	24961647	2015	51

### 3.4 Contribution of author and co-cited author on SUI surgery

**Fig. 5:**
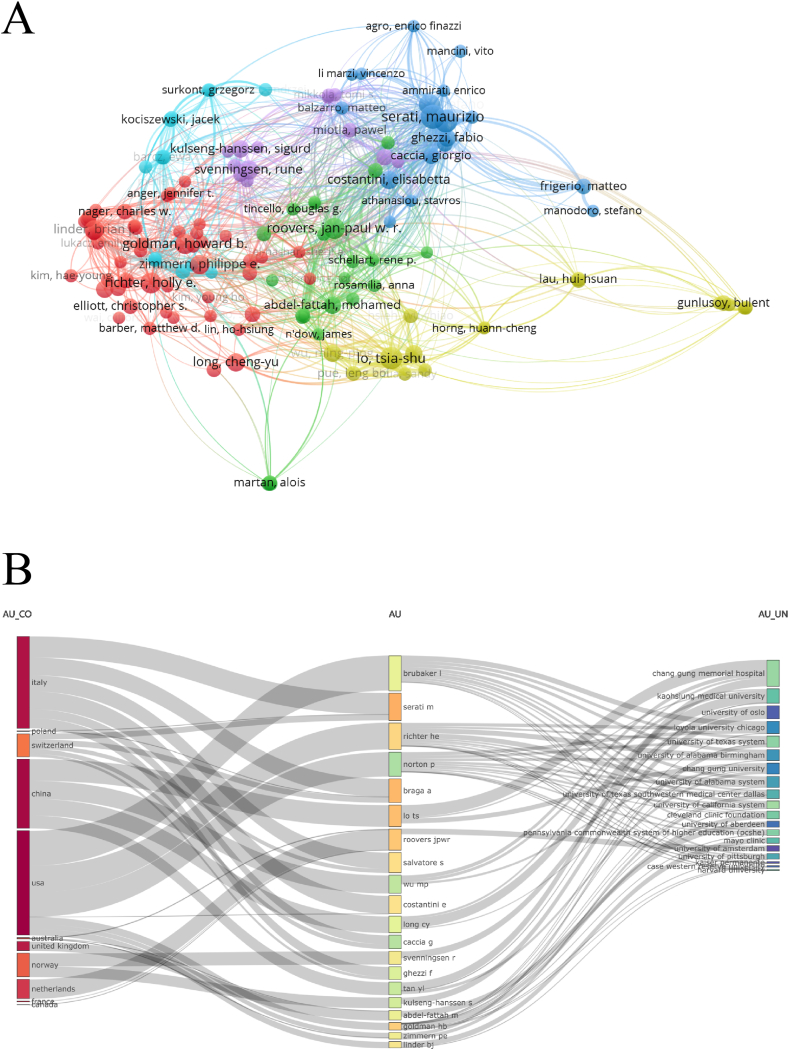
(A) Network visualization map for authors based on the frequency of citations (N = 133, minimum number of citations of one journal ≥5); (B) Mulberry diagram of the relationship between authors, institutions, and countries.

3907 scholars were involved in the field of SUI Surgery and 133 contributed more than 5 papers. The most prolific scholar over the ten years was Serati Maurizio with 19 relevant publications and 242 simultaneous citations. In addition, the scholars who received the most citations and co-citations were Richter Holly E (334) and Ulmsten U (304), respectively. We can see that there is a considerable overlap between the top scholars in terms of number of publications, citations and co-citations, such as Serati Maurizio, Lo Tsia-Shu, and Richter Holly E, which may be the leading scholars in the field (Table 5, Fig. 5A & Sup Fig. 1). And when country, institution and author are analyzed jointly, similar results to those above are obtained, with most of the top scholars concentrated in USA, Italy and China, and closely related to several institutions in the field as mentioned above (Fig. 5B).

**Table 5:** The top 10 authors with the largest numbers of documents and the highest citations, and the top 10 co-cited author with the highest co-citations.

Rank	Author	Documents (n)	Author	Citations	Average Citation	Author	Co-citations
1	Serati Maurizio	19	Richter Holly E	334	23.86	Ulmsten U	304
2	Braga Andrea	17	Roovers Jan-Paul W. R	255	18.21	Haylen BT	303
3	Lo Tsia-Shu	16	Serati Maurizio	242	12.74	Abrams P	216
4	Salvatore Stefano	14	Braga Andrea	212	12.47	Abdel-Fattah M	207
5	Richter Holly E	14	Salvatore Stefano	186	13.29	Ford AA	203
6	Roovers Jan-Paul W. R	14	Goldman Howard B	141	10.85	Serati Maurizio	197
7	Goldman Howard B	13	Lo Tsia-Shu	116	7.25	Richter Holly E	197
8	Svenningsen Rune	12	Linder Brian J	107	8.92	Delorme E	193
9	Linder Brian J	12	Zimmern Philippe E	78	6.5	Nilsson CG	175
10	Zimmern Philippe E	12	Svenningsen Rune	68	5.67	Lo Tsia-Shu	163

### 3.5 Network generation and analysis related to SUI surgery

**Fig. 6:**
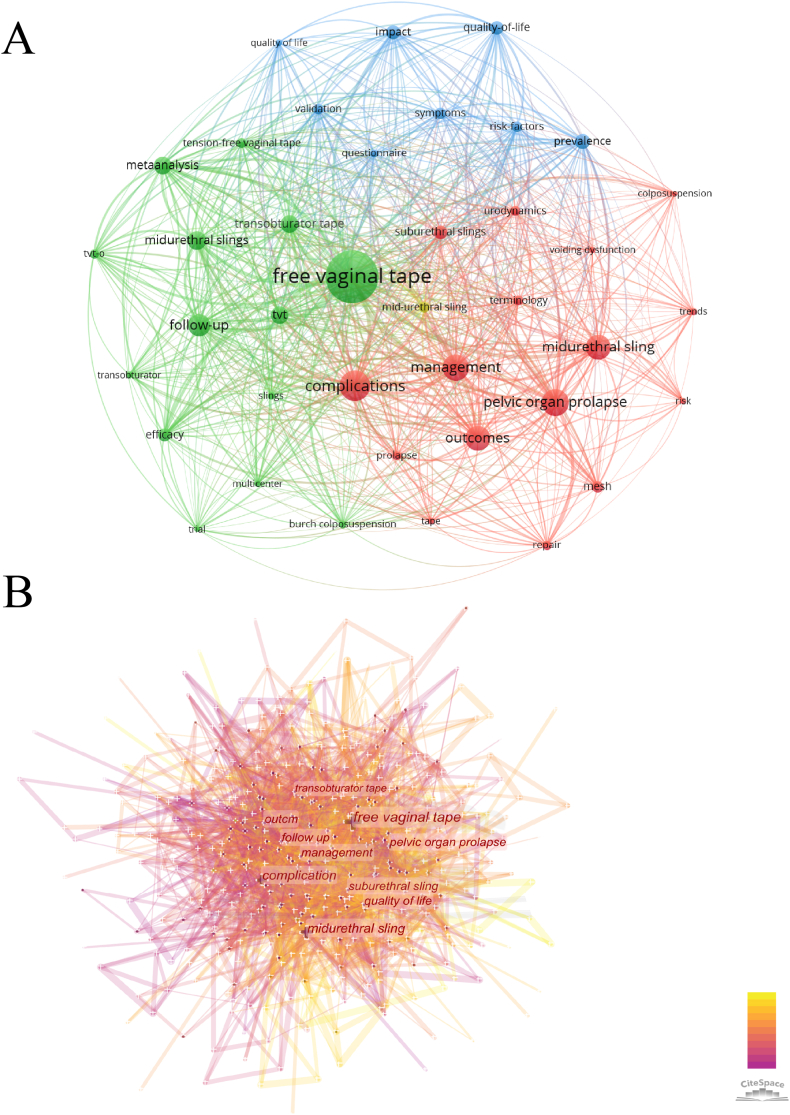
(A) Network visualization map for keywords based on the frequency of occurrences (N = 39, minimum number of occurrences of one keyword ≥30); (B) Analysis of burst words related to SUI surgery.

By analyzing the co-occurrence frequency of keywords, we can gain insights into the research direction and focus of the field. In this analysis, subject words like SUI were excluded. It was observed that the keywords with the highest co-occurrence frequency were free vaginal tape, complications, and pelvic organ prolapse. Furthermore, conducting Citespace burst words analysis revealed similar findings and trends (Fig. 6A and B). When examining the timeline of these keywords and related terms associated with prolapse, it was observed that most of the keywords appeared earlier, indicating a longer time span of research. However, in recent years, terms such as urethral bulking, artificial urinary sphincter, and mesh have emerged, attracting significant attention and becoming hotspots of research (Sup Fig. 2A and B).

## 4 Discussion

SUI, which significantly impacts women's physical and mental health, remains a prevalent issue. While non-surgical methods like Pelvic Floor Muscle Training and electrical stimulation therapy can treat SUI in its early stages, severe cases often require surgical intervention due to inadequate postnatal rehabilitation awareness among mothers [9]. Although tension-free vaginal tape procedure is considered the gold standard for surgical treatment of SUI, the procedure is still associated with numerous complications. To better understand the current development and research status of SUI surgery, as well as identify research hotspots and future trends, we conducted a comprehensive bibliometric analysis of the literature published in the field between 2013 and 2023.

During the ten-year period, the number of annual publications is relatively stable, showing a slow decreasing trend until 2019, but then starting to rise gradually after 2019, which indicates that research related to SUI surgery is re-entering the scholars' field of vision, and one possible reason is that mesh-related complications and bulking agents are gradually regaining attention. However, the amount of about one hundred publications per year is far from enough. The reason may be due to the lack of breakthrough research progress in the surgical treatment of SUI, which stimulates the emergence of a large number of studies, and the slow progress in the field due to the long-term lack of SUI surgical models. Not only that, it is easy to see from the literature search that the vast majority of high-impact studies are clinical cohort studies, which have a long lead time and therefore less output of high-quality studies.

In terms of the countries where the literature has been published, the research centers in this field are mainly located in developed countries and developing countries with higher economic levels. The United States, as the country with the largest number of publications during the decade, six of the top ten institutions in terms of publications are from the United States, and the quality of journals, scholars, and articles are among the top in the world, not only that, but the United States collaborates closely with the world, especially with the Western countries. All evidence clearly shows that the United States is in the lead in SUI surgery research and will remain in the lead for some time.

Meanwhile, China contributes the second largest number of publications in the world, but the average number of citations ranks last among the top ten countries in terms of the number of publications, not only that, there are only two institutions among the top ten in terms of the number of publications and both of them are concentrated in Taiwan Province of China. Epidemiological studies have shown that the prevalence rate of SUI among adult women is about 22.2 %, which suggests that there are a large number of patients with SUI in China, therefore, Chinese scholars should further make full use of the massive patient resources and increase the research on SUI surgery [10]. Not only that, they should also pay more attention to international cooperation, improve the quality of research while focusing on the output, and gradually increase the influence in the field.

In addition, based on bibliometric analysis, we analyzed the ten most cited articles, such as the most cited article is *Sling surgery for stress urinary incontinence in women: a systematic review and metaanalysis*, by Megan O Schimpf et al. published in the American Journal of Obstetrics and Gynecology in 2014, which systematically compared midurethral slings, Burch, and other common surgical procedures for SUI, taking into account objective versus subjective cure rates and taking into account adverse events, and provided an effective tool to a valid tool to assist in clinical decision making [11].

In addition, two randomized controlled trials of retropubic and transobturator mid urethral slings were conducted with a 5-year follow-up, and the results of the two studies were basically the same, with both having good outcomes and satisfaction, and the complication rate remaining low, which suggests that both types of sling surgery have a good therapeutic value and are a good choice for clinical decision-making [12,13].

Interestingly, newer publications have increased interest in adverse events of SUI surgery, especially mesh exposure. This is consistent with our keyword and outbreak word analysis. Joanne R Morling et al. showed a lower risk of immediate complications and subsequent prolapse surgery with mesh surgery compared to major non-mesh surgery, and a similar risk of late complications and further incontinence surgery [14].Kim Keltie et al. showed that patients who underwent surgical Patch implantation for the treatment of SUI 9.8 % of patients experienced perioperative complications within 30 days or 5 years of the initial patch implantation procedure, including but not limited to bleeding, organ perforation, mesh exposure, infection, and pain [15]. Overall, however, the risk of secondary surgery for specific complications was low among adult women undergoing mesh surgery for SUI (10-year cumulative incidence of 3.29 %). However, it is important to note that patients treated by surgeons with lower volumes were 37 % more likely to require surgery for mesh complications; therefore, it is important to standardize the training system for SUI surgery to ensure that surgeons have a high volume of procedures to minimize adverse events due to the skill level of the surgeon [16].

In addition, keywords such as urethral bulking were also found to be of great interest in recent years when keyword-related analysis was performed. Various urethral fillers such as polydimethylsiloxane-Urolastic and hydrogel materials [17,18]. A team also cleverly used microneedle arrays for the treatment of SUI, vigorously pursuing minimally invasive treatment to minimize patient pain [19].

## 5 Conclusion

Overall, this paper combines WOS and related software to provide a bibliometric analysis of publications related to SUI surgery for the period 2013 to 2023, including an assessment of the contributions of countries, institutions, and authors to the field. Our study found that the field has gradually begun to receive attention again in recent years, and that the United States leads the field in terms of both the number of publications and the quality of research. More importantly, our study shows the most cited papers in the field and provides keywords and outbreaks, such as free vaginal tape, which remains a hot topic after many years of research, and complications and mesh, which are emerging as newer research topics. In conclusion, we hope that this paper will provide researchers with a way to get a quick overview of the field and provide ideas for future research.

## Take-home message

Our study provides a comprehensive quantification and summary of publications on stress incontinence surgery over a ten-year period. The study fills a gap in bibliometrics in this field, also avoids wasting research resources and provides lessons for future research.

## Funding

This work was financially supported by Hubei provincial Key Research and Development Program (2022BCA045); The 10.13039/501100012166National Key R&D Program of China: The Establishment of Comprehensive Network for the PFD Prevention, Rehabilitation, Pelvic Floor Surgery and Related Complications (2021YFC2701302; 2021YFC2701303); 10.13039/501100001809National Natural Science Foundation of China (81971364); Second level fund of the second medical leading talents project of Hubei province (no. [2019]47).

## Data availability statement

The original contributions presented in the study are included in the article/Supplementary material, further inquiries can be directed to the corresponding author.

## Ethics statement

All authors have reviewed and agreed to publish the manuscript. Review or approval by an ethics committee was not needed for this study because this manuscript does not involve any animal or human related experiments.

## CRediT authorship contribution statement

**Shufei Zhang:** Writing – original draft, Visualization, Conceptualization. **Jianfeng Liu:** Writing – review & editing, Resources. **Shasha Hong:** Writing – original draft, Investigation, Funding acquisition. **Lian Yang:** Supervision, Resources. **Hanyue Li:** Investigation. **Li Hong:** Writing – review & editing, Funding acquisition.

## Declaration of competing interest

The authors declare that they have no known competing financial interests or personal relationships that could have appeared to influence the work reported in this paper.
