# Supplementary material for: Bibliometric analysis of studies on stress urinary incontinence surgery
Source: Heliyon. 2023 Nov 4;9(11):e21833. doi: 10.1016/j.heliyon.2023.e21833 (PMC10663906; doi:10.1016/j.heliyon.2023.e21833)
Supplement: Multimedia component 1 [file mmc1.docx]

**Bibliometric analysis of studies on stress urinary incontinence surgery**

**Supplementary Material**


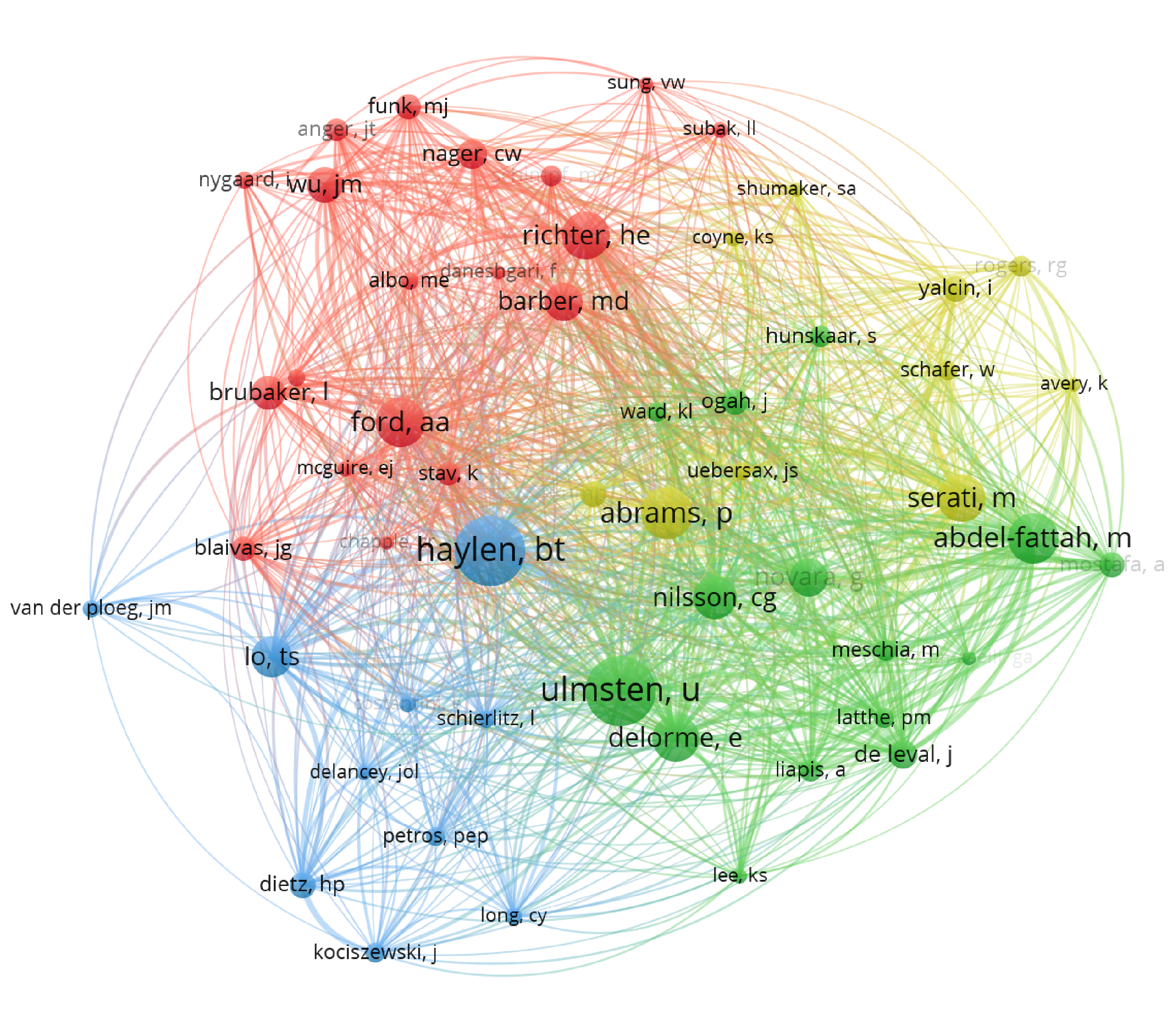


**Supplementary Figure 1.** Network visualization map for authors based on the frequency of co-citation (N = 54, minimum number of citations of one author ≥ 50).


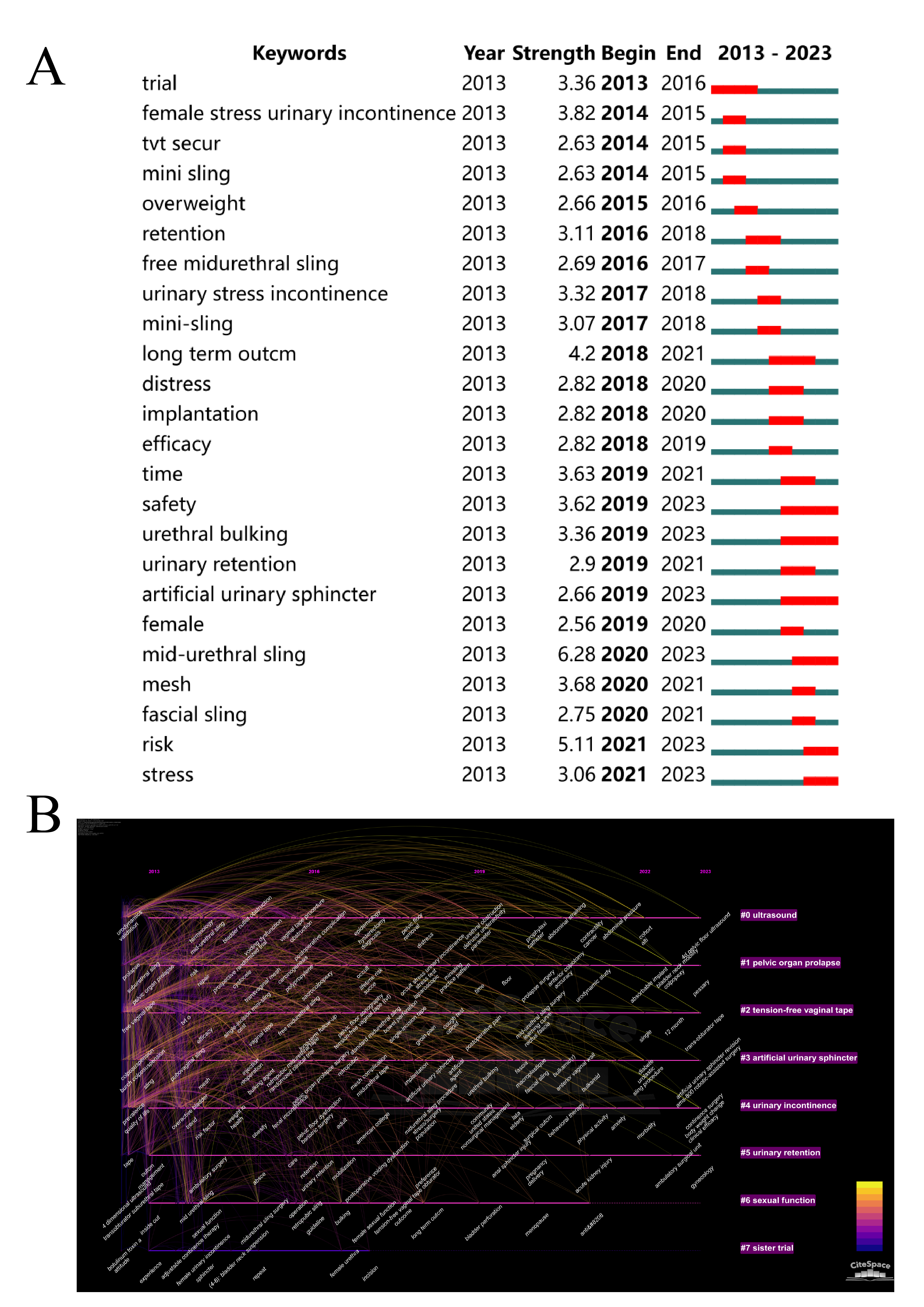


**Supplementary Figure 2.** (A) Top 24 keywords with the strongest citation bursts; (B) Keywords timeline chart for SUI surgery related publications.
